# Cementless total hip arthroplasty for failed treatment of subtrochanteric fracture

**DOI:** 10.1186/s12891-021-04268-8

**Published:** 2021-04-24

**Authors:** Sheng-Yu Jin, Jing-Yao Jin, Min-Gwang Kim, Woo-Jong Kim, Taek-Rim Yoon, Kyung-Soon Park

**Affiliations:** Department of Orthopedic Surgery, Center for Joint Disease Chonnam National University Hwasun Hospital, 322, Seo Yang-Ro, Hwasun-Eup, Hwasun-Gun, Jeonnam 519-809 Republic of Korea

**Keywords:** Subtrochanteric fracture, Failed treatment, Cementless total hip arthroplasty, Complication, Taper stem

## Abstract

**Background:**

Failed treatment of subtrochanteric fractures commonly leads to pain, limping, and poor limb function. Cementless total hip arthroplasty (THA) could serve as an efficient salvage procedure in such cases. This study aimed to evaluate the outcomes and complications of salvage THA in failed subtrochanteric fracture fixation cases.

**Methods:**

From January 2001 to December 2017, cementless THA for failed treatment of subtrochanteric fractures was performed in 18 hips of 11 men and 7 women (average age, 74 years; age range, 57.0–89.0 years). Patients were followed up for clinical and radiological assessments in terms of implant survival and complications after a minimum follow-up of 2 years. The Wagner femoral stems (Zimmer, Warsaw, USA) were used in all 18 patients (100%), with the long-length stem (Wagner SL stem) and standard-length stem (Wagner cone stem) used in 11 and 7 patients, respectively.

**Results:**

The mean follow-up period was 5.2 years (range: 2.2–10.8 years). The mean Harris hip score (HHS) was 38.2 (range: 24–56) preoperatively and 85.4 (range: 79–92) at the last follow-up. The mean postoperative limb length discrepancy was 6.4 mm (range: 4–9 mm). Only one patient underwent revision due to bone in-growth failure of the femoral stem. One patient had an episode of postoperative dislocation and was treated with closed reduction without reoccurrence. Delayed union of the fracture site occurred in one patient. Patients who were previously treated with an intramedullary nail had a significantly shorter surgical duration, lesser intraoperative blood loss, and fewer blood transfusions than those who were previously treated with plate and screws. Kaplan–Meier survival rate with an endpoint of revision was 94.4% (95% confidence interval 72.7–99.9) at 5 years.

**Conclusion:**

Our results indicate that cementless THA is a beneficial and effective procedure for salvaging the failed treatment of subtrochanteric fractures. The Wagner conical prosthesis has shown satisfactory function outcomes, stable fixation, and survival rate for these complex situations. However, attention should be paid to increased operation time, blood loss, and complications when performing THA for subtrochanteric fractures with failed fixation devices especially, plates and screws.

## Background

Subtrochanteric fractures occur in the proximal femur between the inferior aspect of the lesser trochanter and a distance that extends approximately 5 cm distal to it. These types of fractures commonly occur in the younger population (severe comminuted and displaced fractures), caused by high energy impacts, as well as in the elderly population with osteoporosis, caused by low energy impacts (typically long spiral fractures). There are some successful treatments for subtrochanteric fractures that have been introduced in the past decades, including intramedullary fixation and extramedullary plates [1–3]. However, due to the great stress concentration in the subtrochanteric region and high tension across the subtrochanteric area due to various deforming forces, the treatment of these types of fractures is rather challenging [4]. Especially when the fracture pattern is combined with comminuted posteromedial cortex, it shows a high rate of fixation failure, which in turn contributes to a still higher risk of nonunion [5].

The failure of subtrochanteric fracture treatment is frequently associated with unstable fracture patterns, poor bone quality, or suboptimal position of the fixation devices. Such complications following treatment failure may result in nonunion, leading to pain or malunion, severe bone defects, infection, articular cartilage damage, and even post-traumatic arthritis of the hip, which poses a significant challenge subsequent reoperation.

Several salvage treatments can be performed for patients with failed treatment of subtrochanteric fracture, such as re-osteosynthesis [6] and prosthetic replacement [7]. In young patients, efforts should be made to prioritize bone stock preservation through refixation, whereas for elderly patients with inadequate bone stock, hip replacement may be the preferred option [8]. Some studies have reported that total hip arthroplasty (THA) is an effective salvage procedure and can reduce the need for subsequent reoperations for failed treatment of trochanteric fracture fixation [9–15].

However, to the best of our knowledge, few reports have described the results of cementless THA performed as a salvage therapy for failed subtrochanteric fracture fixation. Therefore, the purpose of this retrospective study was to evaluate the clinical outcomes, implant survival, rate of revision, and complications of salvage THA in failed subtrochanteric fracture fixation cases.

## Methods

The institutional review board of our hospital approved this retrospective study. Twenty-two hips of 22 patients with failed fixation of subtrochanteric fractures were treated at our institution between January 2001 and December 2017. One patient (one hip) was lost to follow-up, and three patients (three hips) died due to causes unrelated to THA before 2 years of follow-up; finally, 18 hips of 18 patients who were followed up for more than 2 years were included in this study. Before the operation, infection was preoperatively dismissed in all patients based on complete blood white cell count, total leukocyte count, erythrocyte sedimentation rate, and C-reactive protein levels. There were occurrences of fracture nonunion in all patients, which were related to metal failure in nine patients. In other patients, metal failures were related to metal breakage in eight patients and a cut-out screw in one patient. Prophylactic antibiotics were administered to all patients 30 min before the operation and continued postoperatively for 2 days. Patient demographic details are shown in Table 1.

**Table 1:** The demographic details of the patients

Variable	Value
Male/Female	11/7
Age at time of fracture (years)	72 (range 57.0–86.0)
Age at THA (years)	74 (range 57.0–89.0)
ASA score	1.8 (range 1–3)
Body mass index (kg/m^2^)	23.2 (range 14.6–30.6)
Average follow-up (years)	5.2 (range 2.2–10.8)
Average time to the salvage THA (months)	19.8 (range 4.7–53.8)
Indication	
Nonunion	18
Cutting out / Metal breakage	1 / 8
Fixation Used	
Intramedullary nail	8
Plate device	10
DHS	6
Angled blade plates	2
PF-LCP	1
DCP	1
Frequency of operation before THA	
1/2/3/4	4/12/1/1

Date in gender column, indication, fixation, and frequency of operation columns represent the total number of patients. Data in other columns show an average of the patients within that group. THA is total hip arthroplasty. ASA is the American Society of Anesthesiologists. DHS is dynamic hip screw. PF-LCP is proximal femur locking compression plates. DCP is dynamic compression plate

### Operative technique

All cases of fixation failures were treated using cementless THA by an experienced orthopedic surgeon. All THA procedures were performed using the posterolateral approach in lateral decubitus position. An additional skin incision was made or the original skin incision was extended to remove previously implanted fixation devices when necessary.

After components reduction, the proximal fracture fragment was reattached to the proximal femur using a trochanter grip or a trochanter grip plate with cables (Dall Miles, Stryker Orthopaedics, Mahwah, USA), yielding good tension of the abductors and increasing the healing potential. In all cases, bone grafting was performed routinely for nonunion of the proximal femur using the femoral head. Postoperatively, a range of motion exercises and walking with limited weight-bearing was encouraged a day after surgery. Weight-bearing was gradually increased before obtaining radiological confirmation of bone union. Exercises for a range of hip motion and abductor strengthening were encouraged in all patients.

### Implant description

Wagner straight conical femoral stems with rough blasting were designed for cementless fixation in severe bone deformity or dysplasia at the proximal femur. In our study, Wagner stems (Zimmer, Warsaw, USA) were used in all 18 patients (100%), with the long-length stem (Wagner SL stem) and standard-length stem (Wagner cone stem) used in 11 and 7 patients, respectively. Details of acetabular cup and femoral components and additional devices are shown in Table 2.

**Table 2:** Details of Acetabular and Femoral Components

Variable	Value
Acetabular component	
Delta-PF (Lima-Lto, Udine, Italy)	11
Fitmore acetabular cup (Zimmer, Winterthur, Switzerland)	2
G7 acetabular shell (Biomet, Indiana, USA)	2
Maxera™ cup (Zimmer Biomet, Warsaw, USA)	1
MMC cup (Zimmer Biomet, Warsaw, USA)	1
ACCIS cup (Implantcast, Buxtehude, Germany)	1
Femoral component	
Wanger SL stem	11
Wanger cone stem	7
Additional device	
Trochanteric hook plate	4
Dall-Miles cable-grip system	10
Cable	4

### Follow-up

After the operation, all cases were followed up after operation at 1, 3, 6, and 12 months for the first year and every 6 months after that. During the follow-up, ambulatory status and the Harris hip scores (HHS) were recorded. Postoperative radiographs were assessed routinely for prosthesis position and fixation. Limb length discrepancy was measured from radiographs at the last follow-up, using the method described by Woolson et al. [16]. Femoral stem loosening, stem subsidence, and acetabular loosening were evaluated during follow-ups. For the acetabular cup, a complete radiolucent line at the implant-bone interface and fixation-screw breakage were considered as risk signs for loosening of the cementless socket. Stem subsidence was determined by measuring the distance from the most superior point of the greater trochanter to the most prominent point in the superolateral aspect of the stem [17]. Subsidence greater than 5 mm was considered stem migration. Heterotopic ossification was assessed using the Brooker system [18]. The impact of previous fracture fixation types (Intramedullary nail versus Plate) on the clinical outcomes was also analyzed.

### Statistical analysis

Statistical analyses were performed using independent samples t-test or Mann-Whitney U test by the Statistical Package for Social Science (SPSS) software (Base 25.0 SPSS Inc. Chicago, Ill); *P* < 0.5 was deemed significant. Survival of implants was analyzed using the Kaplan–Meier survival curve with 95% confidence intervals (CI). The starting point was the date of salvage THA and endpoint was the date of revision.

## Results

The mean follow-up period was 5.2 years (range: 2.2–10.8 years). There were 11 men and 7 women, with an average age of 74 years (range: 57.0–89.0 years). At the time of previous internal fixation, the mean patient age was 72 years (range: 57.0–86.0 years). Several kinds of previous fracture fixations were used: a intramedullary nail (*n* = 8), a dynamic hip screw (*n* = 6), an angled blade plate (*n* = 2), a proximal femur locking plate (*n* = 1), or a dynamic compression plate (*n* = 1). All subtrochanteric femur fractures were classified based on the Orthopaedic Trauma Association (OTA) system and Russell–Taylor criteria. Among these 18 patients, 13 (72.2%), three (16.7%), and two (11.1%) had type A, B, and C fractures, respectively, based on the OTA classification system. According to the Russell–Taylor classification, subtrochanteric fractures were types 1A, 1B, 2A, and 2B in eight (44.4%), three (16.7%), four (22.2%), and three (16.7%) patients, respectively. The mean interval between primary fixation and salvage THA was 19.8 months (range: 4.7–53.8 months).

All fracture cases remained unhealed after one to three surgical treatments. Twelve patients had undergone revision fixation twice with bone grafting before conversion THA; four patients with failed fixation of a subtrochanteric fracture underwent no revision fixation owing to inadequate bone quantity; the other two patients underwent three and four revision fixation surgeries, respectively.

The mean duration of the THA operation was 98 min (range: 70–135 min). The mean total blood loss was 992.2 mL (range: 640–1260 mL), and the amount of blood transfused was 2.2 units (range: 1–4 units). The mean HHS was 38.2 (range: 24–56) preoperatively and 85.4 (range: 79–92) at the last follow-up. The mean postoperative HHS of patients with subsidence of stem was lower than that of patients without postoperative subsidence of stem, but it was not significantly different. The mean postoperative limb length discrepancy was 6.4 mm (range: 4–9 mm).

Before the salvage operation, all cases (100%) had a walking disability; they had to use crutches because of hip pain. After the salvage THA, nine patients (50%) could walk without assistance, eight (44.4%) could walk using a cane, and one (5.6%) could walk using a single crutch. Subsidence of the stem occurred in nine patients (50.0%) 3 months after surgery. In this case series, the mean subsidence value was 3.4 mm in patients. One hip (5.6%) subsided more than 5 mm, which required stem revision; this case underwent previous intramedullary nailing refixation before conversion THA.

**Fig. 1:**
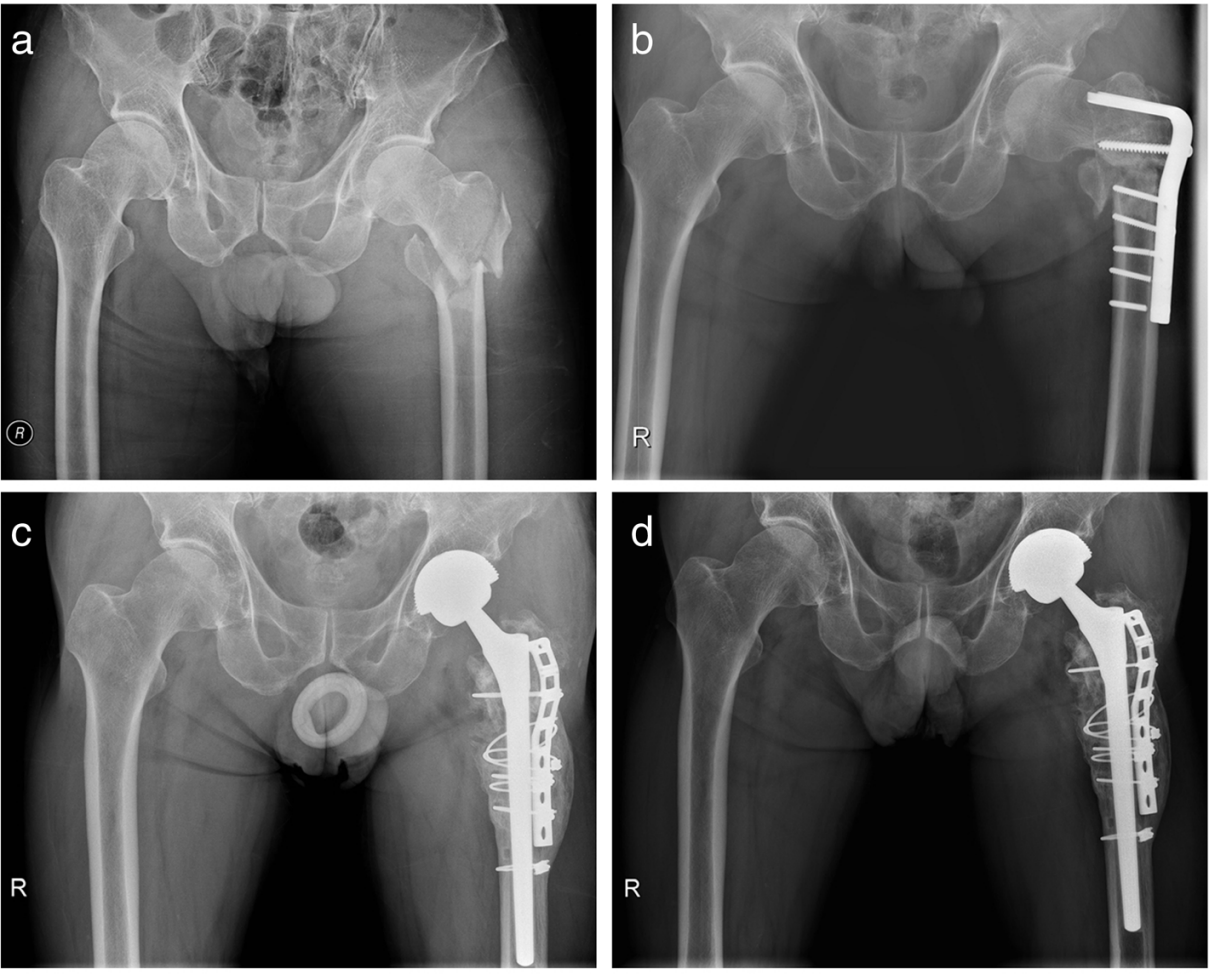
**a** A 69-year-old man sustained a subtrochanteric fracture of the left femur. **b** Preoperative radiograph showing the failure of the angled blade plate with screw breakage and nonunion of subtrochanteric fracture 15 months after internal fixation. **c** The plate was removed and total hip arthroplasty was performed with long femoral stem and reconstruction of fragments with the cable grip system. **d** Six years postoperatively, the patient returned to unassisted ambulation with a pain-free range of motion of the hip joint

**Fig. 2:**
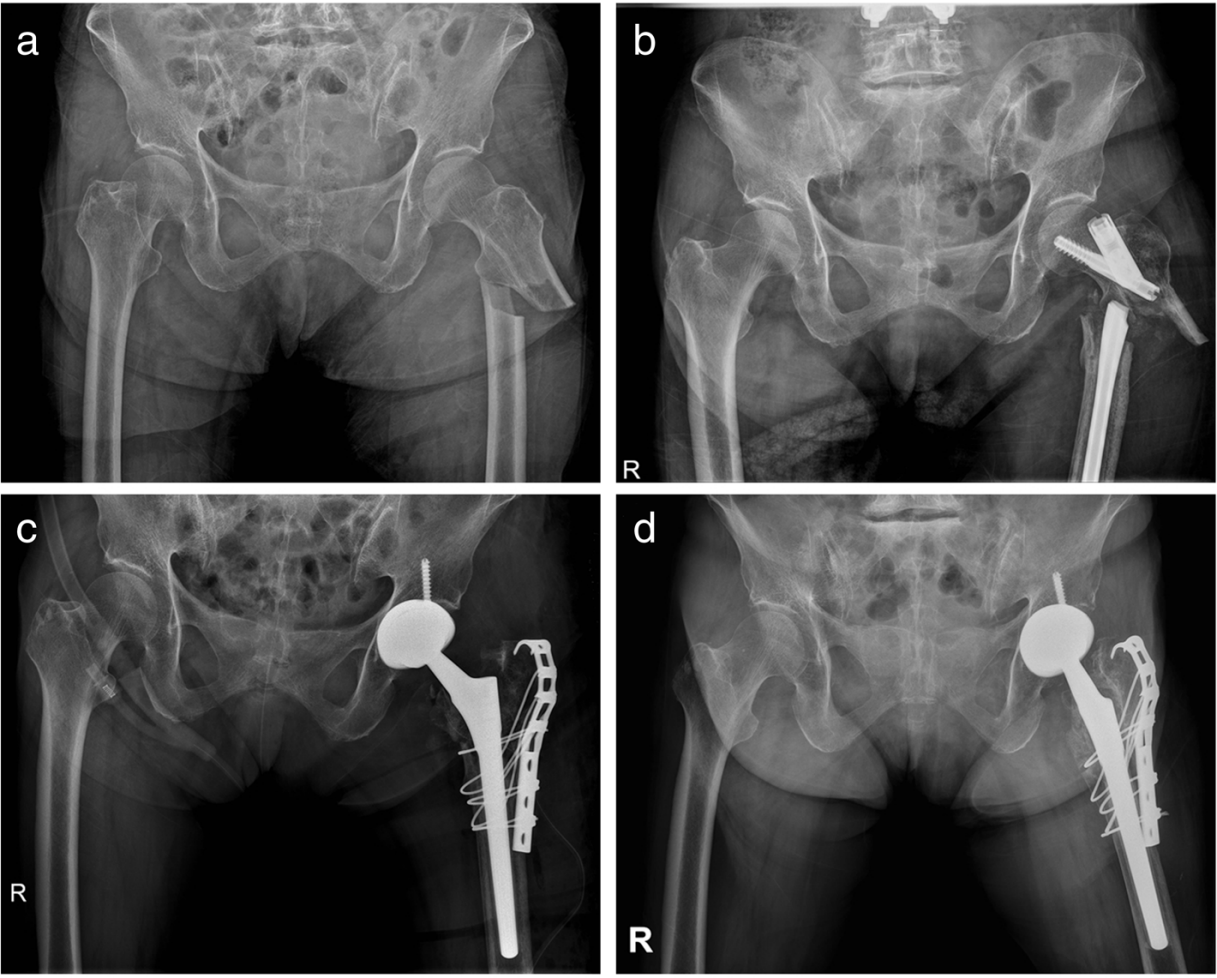
**a** A 72-year-old woman sustained an atypical femur subtrochanteric fracture of the left femur. **b** The nail breakage and nonunion of subtrochanteric fracture 13 months after internal fixation. **c** The device was removed and total hip arthroplasty was performed with long femoral stem and trochanteric reattachment using a cable grip system. **d** Two years and eleven months postoperatively, the patient with remarkable pain relief and return to ambulation

In the group of patients previously treated with plate fixation, there were five cases with metal breakage of the device (Fig. 1), whereas, in the group that underwent previous fixation with intramedullary nails, there were three cases (Fig. 2). Patients with a previous intramedullary nail fixation had a shorter surgical time, fewer blood transfusions, and less intraoperative blood loss than those previously with plate fixation (Table 3).

**Table 3:** The subanalysis of clinical results on fixation types

Variable	Plate	Intramedullary nail	*P*-value
Number of hips	10	8	/
Operation time (minutes)	106.9 (95–135)	86.9 (70–100)	0.004
Estimated blood loss (mL)	1099.0 (900–1260)	858.6 (630–960)	0.001
Transfusion (number of units)	2.6 (2–4)	1.8 (1–3)	0.021
Hospital stay (days)	26.2 (14–36)	26.0 (13–44)	0.981

### Implant survival

**Fig. 3:**
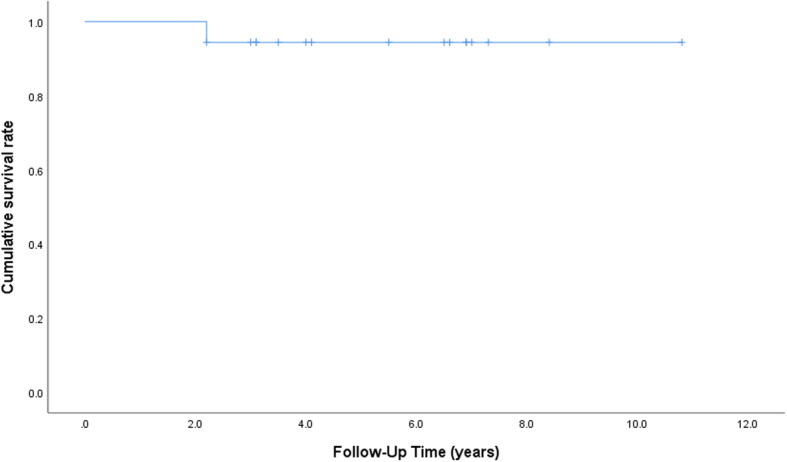
Kaplan–Meier curve, with 95% confidence interval for revision of the implant for any

The Kaplan–Meier survival analysis, with revision of the implant for any reason as the endpoint, revealed a survival rate of 94.4% (95% CI: 72.7–99.9) at 5 years (Fig. 3). Only one revision due to bone in-growth failure of femoral stem was performed, and this patient underwent revision with a cemented stem (CPT, Zimmer, Warsaw, USA).

### Complications

Complications occurred in two of the 18 hips (11.1%). One patient had a postoperative dislocation and was treated by closed reduction with no reoccurrence. One patient showed delayed union of the proximal fracture site, which is defined as failure to reach bony union by 6 months after THA. No patients underwent reoperation even for the removal of trochanter grip or grip plate. For the cementless cups, no malposition or aseptic loosening was detected. There were two cases with class I heterotopic ossification and one patient with class II heterotopic ossification.

## Discussion

Salvage treatment for failed subtrochanteric fracture fixation is challenging. Re-osteosynthesis with internal fixation or plates and conversion to hip replacement is accepted surgical salvage options. Elderly patients with metal failure of subtrochanteric fractures usually have poor bone quality in the proximal femur. In this situation, refixation will prolong their bed-rest period and prevent early ambulation, often necessitating replacement surgery [8]. Some previous studies have reported that THA could provide a higher level of function and favorable results than hemiarthroplasty [19, 20]. We also preferred THA over hemiarthroplasty because patients often experience pain associated with acetabular cartilage destruction after hemiarthroplasty. Besides, in our series, most patients with failed fixation had experienced long-term non-weight-bearing conditions of the hip joints, which could lead to poor cartilage conditions.

Several authors have reported the results of THA in failed fixation of proximal femur fracture patients [7, 9–15, 20, 21]. Most of these studies focused on failed fixation of intertrochanter fracture but fewer studies on the subtrochanteric fracture. In a series of 88 patients treated with a salvaged hemiarthroplasty or THA after failed fixation of trochanteric or subtrochanteric fractures [7], standard femoral stems or long stems were used in all patients. During a 5–11 year of follow-up duration, the revision rate was 16% (14/88 hips). Using long length stems could reduce the risk for reoperation due to bridge previous bone defects. Weiss et al. [22] reported 30 salvaged THAs using a modular cementless stem for failed trochanteric or subtrochanteric fractures with a mean follow-up period of 4 years. Twenty patients from their study had subtrochanteric fractures. Revisions were conducted for one case of recurrent dislocation, five cases of infection, one case of periprosthetic fracture, and the rate of reoperation was 23% (7/30 cases). In our study, most of the cases showed good pain relief and ambulated independently with or without support, consistent with previous findings from other published literature [7, 10, 15, 22]. Besides, most of the proximal femur fractures were united, except for one delayed union. Only one patient received stem revision due to bone in-growth failure.

In our series, we found that patients previously treated with an intramedullary nail had a significantly shorter surgical duration, lesser intraoperative blood loss, and fewer blood transfusions than those previously treated with plate and screws. We believe that intramedullary nails might be a better initial implant of choice for the treatment of subtrochanteric fractures, particularly as they can easily be converted to THA in cases of failure. When performing salvaged THA for subtrochanteric fracture with failed external plate fixation, we should pay special attention to increased operation duration, blood loss, and complications.

Dislocation is another severe problem seen after the salvage procedure, often as a result of poor hip joint stability. Zhang et al. [23] reported that the overall dislocation rate of rescue hip replacement after the failure of proximal femoral internal fixation was 15.8%. Archibeck et al. [24] reported that dislocation occurred in five of 102 patients who treated with conversion THA, and reoperation was performed in one patient because of instability. However, only closed reduction was required for one patient in this study, which can be attributed to the well-orientated position of the components and sufficient repair of the short external rotators and abductor mechanism reconstruction. In the present study, four patients with failed fixation of a subtrochanteric fracture did not have undergone revision fixation due to insufficient bone stock. It was challenging and unlikely for severe bone loss to obtain sufficient fixation and guarantee union through re-osteosynthetic surgery alone.

In the present study, the Wagner femoral stems (Zimmer, Warsaw, USA) were used in all patients. The Wagner conical stem is a tapered, fluted, grit-blasted cementless femoral prosthesis. This femoral stem was designed for treating complex primary THA or revision cases. Sandiford et al. [25] reported 104 femoral revisions using the Wagner SL stem, with a follow-up of 24–46 months and found no femoral stems had failed due to aseptic loosening; only one patient with the stem and cup infection required operation [25]. Previous studies have shown that the Wagner conical femoral stems have excellent survival rates and favorable results in revision and complex primary THA [26, 27], potentially making it a better choice. The Wagner stem showed good implant survival in the present study, with only one stem revision due to the bone in-growth failure.

Subsidence of the femoral stem could be a common complication in cementless stems and can lead to osseointegration failure, especially in complex THA. In this study, nine patients (50%) showed stem subsidence within 3 months of THA, but the amount of stem subsidence had no influence on clinical outcomes, such as thigh pain, HHS, or patient satisfaction. The subsidence rate observed in this cohort is comparable to previously reported rates of subsidence for femoral stems [28, 29]. Of the cases included in this study, only one stem was revised because the previous instrumentation had negatively impacted the medullary canal and femoral stem fixation. Great care should be taken to avoid undersize the cementless femoral stems selection during operation.

There are some limitations to this study. First, we did not set up a control group to compare the outcomes, and the study was retrospective. Second, there were a limited number of sample cases, and the follow-up duration was also relatively short. However, to the best of our knowledge, only two other studies have reported on more than 18 cases of failed THA for subtrochanteric fractures [7, 22] and ours appears to be the first study to evaluate the effect of a salvage cementless THA using the Wagner stem for failed subtrochanteric fracture. Third, the patients included in this study were operated on for rare diseases with different types of cups for a long period of up to 16 years, which could prove to be a confounding factor for this study.

## Conclusion

Our results indicate that cementless THA is a beneficial and effective procedure for salvaging the failed treatment of subtrochanteric fractures. The Wagner conical prosthesis has shown satisfactory function outcomes, stable fixation, and survival rate for these complex situations. However, attention should be paid to increased operation time, blood loss, and complications when performing THA for subtrochanteric fractures with failed fixation devices especially, plates and screws. We believe that this report conveys valuable messages to surgeons who suffer technical difficulties following failed subtrochanteric femur fractures.

## Data Availability

The data sets supporting the results of this article are included within the article and its additional files. The datasets are available from the corresponding author on reasonable request.

## Abbreviations

THA: Total hip arthroplasty
SD: Standard deviation
HHS: Harris hip score
OTA: Orthopaedic Trauma Association
CI: Confidence intervals
